# Supplementary material for: A versatile coherent Ising computing platform
Source: Light Sci Appl. 2026 Jan 20;15:74. doi: 10.1038/s41377-025-02178-1 (PMC12816571; doi:10.1038/s41377-025-02178-1)
Supplement: Supplementary file 1 — Supplemental Material [file 41377_2025_2178_MOESM1_ESM.pdf]

Supplementary Information for

A versatile coherent Ising computing platform

Hai Wei[1*], Chengjun Ai[1], Putuo Guo[1], Bingjie Jia[1], Lixin Yuan[1], Hanquan Song[1], Shaobo Chen[1], Chongyu Cao[1], Jie Wu[1], Chao Ju[1], Yin Ma[1,2], Jintao Fan[3], Minglie Hu[3], Chuan Wang[1,2*], Kai Wen[1*]

[1] Beijing QBoson Quantum Technology Co., Ltd., Beijing, 100016, China

[2] School of Artificial Intelligence, Beijing Normal University, Beijing, 100875, China

[3] Ultrafast Laser Laboratory, School of Precision Instruments and Opto-electronics Engineering, Tianjin University, Tianjin, 300072, China

*Corresponding author. Email: weih@boseq.com, wangchuan@bnu.edu.cn, wenk@boseq.com

1. QUBO problem for the Ising model

The QUBO problem can be generally expressed as follows [1]:

$$f = \sum_{i,j=1}^{n} Q_{ij} x_i x_j + \sum_{i=1}^{n} w_i x_i \tag{1}$$

where $x_i = 0,1$. When $i = j$, $x_i x_j = x_i^2 = x_i$, thus yielding:

$$f = \sum_{i,j=1}^{n} Q_{ij} x_i x_j + \sum_{i=j=1}^{n} w_i x_i x_j \tag{2}$$

The term w_i can be incorporated into the diagonal elements of Q_{ij} to yield the following formula:

$$f = \sum_{i,j=1}^{n} Q'_{ij} x_i x_j \tag{3}$$

Here, Q' represents the combined matrix of Q_{ij} and w_i.

The general form of the Ising model is given by:

$$H = \sum_{i,j=1}^{n} J_{ij} s_i s_j + \sum_{i=1}^{n} \mu_i s_i \tag{4}$$

The Ising model can be transformed into the QUBO problem by substituting $s_i = 2x_i - 1$ into Equation(4). Conversely, replacing $x_i = \frac{s_i + 1}{2}$ in the QUBO problem allows for the conversion to the Ising model. Currently, our fs CIM only supports the Ising model without Zeeman terms, as shown in Equation (12). The Ising model in with Zeeman terms (Equation(4)) can be transformed into Equation(5) by introducing additional variable s_{n+1} using Kaiwu SDK [3].

$$H = \sum_{i,j=1}^{n} J_{ij} s_i s_j \tag{5}$$

2. **The cloud service platform and Kaiwu SDK**

The Coherent Photonic Quantum Computing Cloud Platform[2] from Beijing QBoson Quantum Technology Co., Ltd., migrates the computing resources of coherent photonic quantum computers to the cloud via standardized interfaces. It delivers sustained, stable, and task-oriented quantum computing services on real hardware to users. Through this cloud platform, users can transform complex combinatorial optimization problems into quantum computing tasks and solve them using quantum hardware. The platform's core operations include problem mapping, resource scheduling, execution of quantum evolution on the CIM computing architecture, and delivery of high-quality solutions. To support these functions, the cloud service comprises the following key components:

Quantum Application Service: Provides users with feasible solutions.

Quantum Development Platform: Handles problem modeling and generates problem matrices for the quantum computer.

Task Management Platform: Manages task scheduling, maintains the quantum computer job queue, invokes quantum hardware for computation, and performs result post-processing.

Security Management: Oversees security-related functions, including identity authentication, access control, and data encryption.

Operations Management: Focuses on core operational tasks such as daily system maintenance, resource scheduling, and system status monitoring.

Kaiwu SDK [3] is a comprehensive software development kit tailored for addressing QUBO problems via CIMs. It offers an intuitive Python-based platform to create algorithms that can be executed on CIM hardware, accompanied by a physical interface. Kaiwu SDK includes a diverse

set of classical solvers, CIM simulation tools, and essential pre-processing and post-processing modules.

3. **XGBoost**

XGBoost is a highly efficient, flexible, and portable distributed gradient boosting library that implements machine learning algorithms within the gradient boosting framework. It offers parallel tree boosting (also known as Gradient Boosted Decision Trees) to efficiently solve various data science problems with speed and accuracy. The code is compatible with major distributed environments such as Hadoop, Sun Grid Engine, and MPI and can handle problems involving billions of examples [4]. In the context of credit scoring, XGBoost offers significant advantages over other algorithms because of its robust predictive capabilities and ability to mitigate over-fitting. Through the iterative construction of weak learners that rectify errors from previous steps, XGBoost develops a powerful integrated model that is well suited for handling complex data and capturing non-linear relationships. Furthermore, by incorporating regularization terms into the objective function, XGBoost effectively manages model complexity, mitigates over-fitting risks, and enhances generalizability.

4. The alignment of optical and electrical signals

The alignment of optical and electrical signals plays a critical role in signal control, consequently impacting computational outcomes. To augment solution quality, our system integrated error control mechanisms for aligning optical and electrical signals. Experimental tests were conducted on a single non-regular random graph instance comprising N = 100 vertices and E = 700 edges, as depicted in Fig. S1. Multiple 100-run batches were executed for this instance.

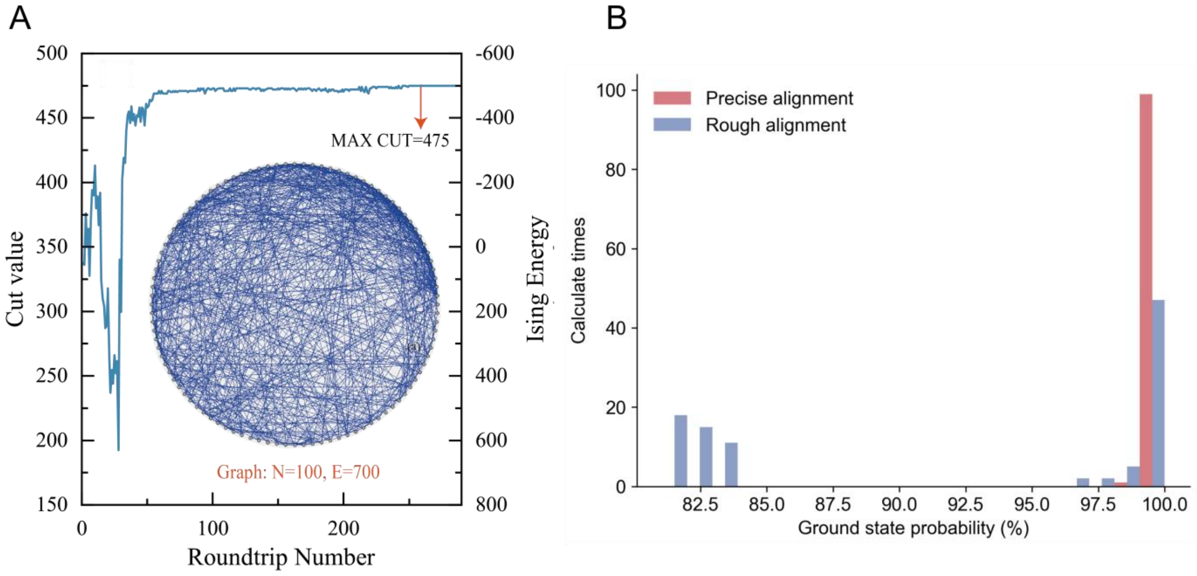

Fig. S1. Results with different alignment of optical and electrical signals. (A) Time evolution of the cut value. (Inset) The graph being solved. (B) Histogram of obtained cut values in 100 runs for the graph shown in the insets of Fig. S1A, when the signals are precisely aligned and roughly aligned.

As demonstrated in Table 2, precise alignment of optical and electrical signals resulted in a success probability of 60% for achieving a ground state. For instances where a ground state was not attained, the system successfully obtained good approximate solutions, with a probability exceeding 98% of achieving a solution within x% of the optimal (maximum cut). Fig. S1 illustrates the solution distribution obtained from 100 runs with precise alignment.

When the signal is precisely aligned, the peak value of the sine wave electrical signal coincides with the signal peak value measured by BHD, and a slight deviation in the electrical signal does

not correspond to the peak value. However, even a slight offset in alignment (approximately 80ps apart, considering a pulse width of 250 ps for the electrical signal) led to a significant drop in the probability of attaining the optimal solution, reducing it to 30%. Notably, precise alignment correlated with a 35% improvement in both the 98% and 95% confidence levels, as depicted in Fig. S1, contrasting with the solution histogram obtained from 100 runs with rough alignment of the photoelectric signal.

In terms of stability, deviations in solution probability and increased standard deviation were observed when alignment was slightly off. Hence, the precise alignment of optical and electrical signals emerges as imperative for achieving higher probabilities of optimal solutions and ensuring solution stability.

5. Energy efficiency of the system

To comprehensively evaluate the performance of the CIM solver in this work, this section presents the energy consumption of the CIM and compares it with that of other classical methods. The total power consumption the CIM device mentioned in this manuscript is approximately 432 W during operation. This includes all modules such as the FPGA, SHG, PSA, and so on. The single computation time is 464 μs for a Möbius ladder graph with 100 vertices, and the total energy consumption per solution is approximately 0.2 J. Digital computing schemes, like the simulated annealing (SA) algorithm, mainly rely on servers. When a server with an AMD EPYC 7763 CPU and an H12DSi-N6 motherboard is used to execute the simulated annealing program, the CPU power during the calculation is 140 W. When using the single-threaded SA to solve the

Möbius graph with 100 vertices, the computation time is about 120 ms. The total energy consumption per solution amounts to 16.8 J.

The power consumption of our CIM is higher than that of traditional CPUs. However, the CIM holds an edge in terms of single computation time. Consequently, in terms of overall energy efficiency, it is better to the SA. In the future, with the continuous optimization of the CIM's performance, for example, if the CIM can operate at a lower pump power, its power consumption is expected to be further reduced.

6. The cavity stability system

In our CIM system, the total cavity length is approximately 420 meters, the vast majority of which is fiber. The free-space segment is very short—less than 2 meters—and accounts for less than 0.5% of the total length. Consequently, the fiber part is far more sensitive to length variations and, therefore, to temperature fluctuations. So, we place the fiber in an incubator and use an active temperature control system to maintain the temperature inside the incubator. But for optical components, we only use acoustic foam to shield them without other spatial temperature control.

For the fiber part, we designed a compact airtight container, with a fiber bobbin placed at its innermost layer. The fiber bobbin is housed in a sealed thick-walled insulated container, around the outer wall of which heat exchange copper tubes are coiled. These copper tubes are filled with water and connected to an active temperature controller via pipelines. The active temperature controller regulates the water temperature with a precision of 0.01℃. Through active

temperature control, the temperature inside the insulated container can be maintained within a fluctuation range of 0.01℃.Subsequently, the copper tubes together with the insulated container are placed inside a Dewar flask. Finally, the Dewar flask, after being packed with foam, is installed in a movable aviation aluminum case. These two outer layers isolate the internal heat exchange copper tubes and the optical fiber container from ambient temperature variations, thereby improving the accuracy of temperature control. The temperature fluctuations are measured and recorded in Table S1.

Time	Temperature fluctuations (/30 minutes)		
	Cavity (fiber)	Cavity (space)	Environment
0~0.5h	0.015	0.098	1.3
0.5~1h	0.009	0.145	1.3
1~1.5h	0.008	0.146	1.4
1.5~2h	0.008	0.138	1.4
2~2.5h	0.01	0.133	1.4
2.5~3h	0.012	0.145	1.1
3~3.5h	0.01	0.068	0.7
3.5~4h	0.01	0.015	0.9

Table S1. Temperature fluctuations (/30 minutes) of the spatial optical path and the surrounding fiber before and after temperature control.

The free-space part is mainly built on an optical table. It has large area and needs to adjust frequently. This makes it difficult to build high-precision temperature control for the entire free-space components. So, we used acoustic foam to protect the free-space part. The acoustic foam can isolate vibrations and, to a certain extent, smooth free-space temperature fluctuations.
For the short-term stability, we use a dither-and-lock scheme to control a piezoelectric transducer (PZT) to adjust cavity length at the sub-micrometer scale. During the computation process, the

auxiliary bits are driven to a stable phase. The dither-and-lock scheme will apply a small dithering signal inside the cavity, which is achieved through the stretching of the PZT. The dithering signal will result in an intensity modulation of the auxiliary bits, and the environmental fluctuation is also superimposed on the auxiliary bits phase. We use a photodiode (PD) to detect the phase of the auxiliary bits. The detected signal undergoes analog-to-digital conversion, followed by demodulation, filtering, and amplification to obtain an error signal that reflects environmental fluctuations. Finally, via the PID algorithm, a control signal is generated based on the error signal and applied to the PZT. to counteract rapid length fluctuations of the cavity.

7. The precision of the FPGA portion

As shown in Figure 1, when the number of vertices of Möbius Ladder problem gradually increases from 20 to 100, the success rate decreases progressively. Here are some extra experiments to check whether the success rate related to the precision of the FPGA portion.

First, we identified potential precision issues in the FPGA processing workflow:

We use an Analogue-to-Digital Converter (ADC) to convert analog signals into digital signals. The ADC has a 14-bit width, which may introduce precision issues.

The calculation of the formula in the FPGA uses fixed-point arithmetic, and this calculation process involves no precision loss.

However, the feedback injection strength of each pulse transmits to the push-pull modulator via a DAC. The DAC also has 14-bit width, so precision issues (such as saturation or truncation) may also occur in this process.

It can be seen that the bit width of the ADC/DAC is the key factor affecting precision. Therefore, we performed extra experiments where we artificially truncated the precision of the ADC and DAC. By using different truncation of bit widths, we equivalently limited the device precision. Under these conditions, we tested the success rate of the 100-bit Möbius Ladder graph respectively.

Based on the results, the success rate may have little correlation with the FPGA precision. As shown in Fig.S2, we set the sampling bit width of the ADC and DAC to 8, 10, 12, and 14 bits, with the corresponding average success rate being 54.8%, 54%, 52.7% and 54.8% respectively. It can be observed that the variation in the average power is extremely small, with only 2%.

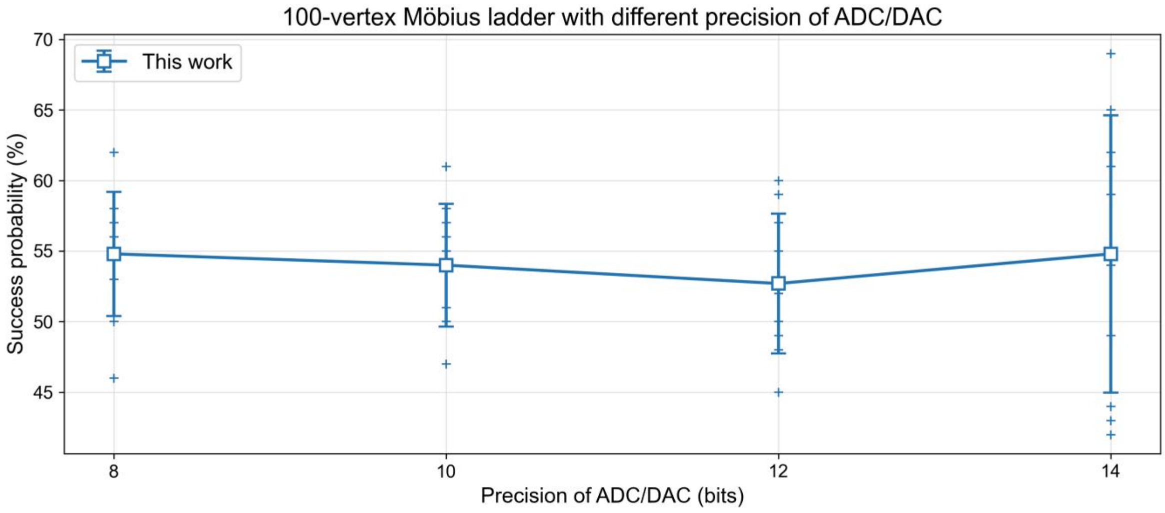

Fig. S2. Max-cut results for 100-vertex Möbius ladder graphs with different precision of ADC/DAC. Measured probability of achieving the ground state in a single run, as a function of number of vertices. Multiple 100-run batches were performed for each graph to obtain the standard deviations, which are shown as error bars.

8. Ligand-protein molecular docking system

The objective of molecular docking is to identify the ligand molecule that can bind most stably to the target protein. Computational simulation-based methods enable rapid screening of active molecules. To evaluate the effectiveness of different computational simulation approaches, the root-mean-square deviation (RMSD) between the ligand conformations obtained computationally and the standard experimental results is frequently calculated. In this study, we selected three protein–ligand systems (1N2J, 1LRH, and 1JD0) in benchmark dataset to validate the performance of the algorithm and computing platform.

1N2J: Crystal structure of a pantothenate synthetase from M. tuberculosis in complex with pantoate.

1LRH: Crystal structure of auxin-binding protein 1 in complex with 1-naphthalene acetic acid.

1JD0: Crystal structure of the extracellular domain of human carbonic anhydrase XII complexed with acetazolamide.

These systems correspond to experimentally determined crystal structures and each of the system requires approximately 110 computational bits after being modeled as a QUBO. Based on the

model, the computed docking poses (pantoate, 1-naphthalene acetic acid, acetazolamide) closely matched the experimental results, indicating our QUBO docking computational model can achieve results comparable to those obtained from experiments.

Reference

1. Takesue, H. *et al.* Finding independent sets in large-scale graphs with a coherent Ising machine. *Sci. Adv.* **11**, eads7223 (2025).
2. Beijing QBoson Quantum Technology Co., Ltd. https://platform.qboson.com/ (2022).
3. Beijing QBoson Quantum Technology Co., Ltd. Kaiwu SDK. https://kaiwu-sdk-docs.qboson.com/en/ (2022).
4. Chen, T. & Guestrin, C. XGBoost: A Scalable Tree Boosting System. *Proceedings of the 22nd ACM SIGKDD International Conference on Knowledge Discovery and Data Mining* 785–794 (2016).
